# Supplementary material for: Dynamic organelle distribution initiates actin-based spindle migration in mouse oocytes
Source: Nat Commun. 2020 Jan 14;11:277. doi: 10.1038/s41467-019-14068-3 (PMC6959240; doi:10.1038/s41467-019-14068-3)
Supplement: Supplementary file 1 — Supplementary Information [file 41467_2019_14068_MOESM1_ESM.pdf]

Supplementary Information

Dynamic Organelle Distribution Initiates Actin-Based Spindle Migration in Mouse Oocytes

Xing Duan *et al*.

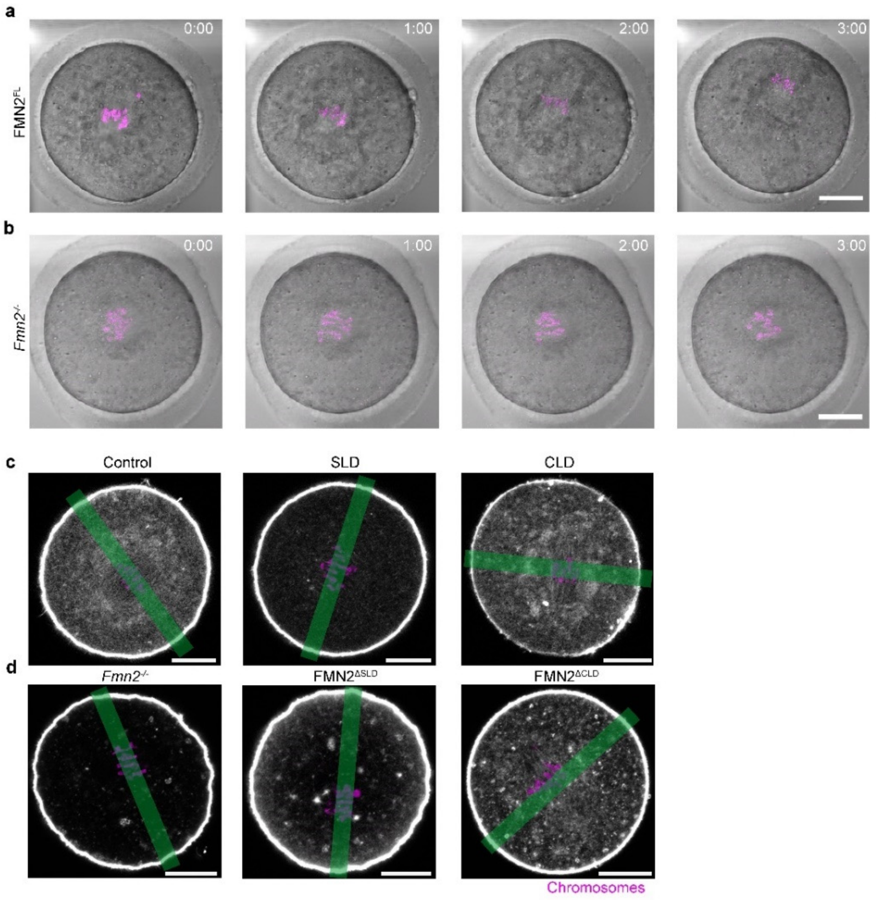

Supplementary Figure 1. FMN2^{FL} rescued spindle migration in Fmn2$^{-/-}$ oocytes and the measurement of cytoplasmic and cortical actin intensity by line trace crossing the individual oocytes.

a, b Representative time-lapse images of chromosomes migration in Fmn2$^{-/-}$ oocytes (**b**) and FMN2^{FL}-expressing Fmn2$^{-/-}$ oocytes (**a**). Magenta, chromosomes. Scale bar, 20 μm. **c,** Images showing the lines for tracing actin intensity in control, SLD and CLD-expressing oocytes. **d**, Images showing the lines for tracing actin intensity in Fmn2$^{-/-}$, FMN2$^{\Delta SLD}$ and FMN2$^{\Delta CLD}$-expressing oocytes in Fig. 2c and d.

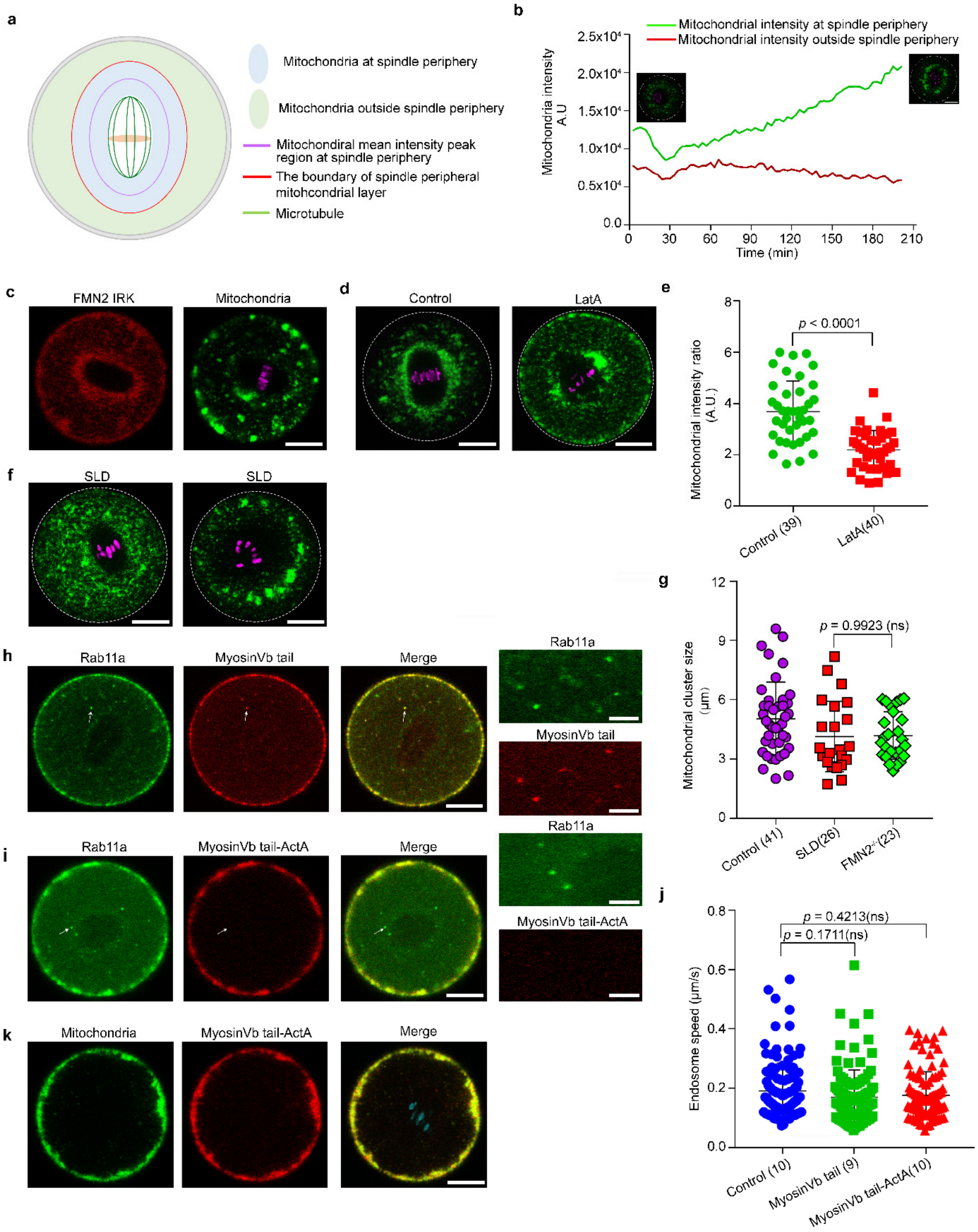
a
Mitochondria at spindle periphery
Mitochondria outside spindle periphery
Mitochondiral mean intensity peak region at spindle periphery
The boundary of spindle peripheral mitohcondrial layer
Microtubule
b
Mitochondrial intensity at spindle periphery
Mitochondrial intensity outside spindle periphery
Mitochondria intensity A.U
Time (min)
c
FMN2 IRK
Mitochondria
d
Control
LatA
e
p < 0.0001
Mitochondrial intensity ratio (A.U.)
Control (39)
LatA(40)
f
SLD
SLD
g
p = 0.9923 (ns)
Mitochondrial cluster size (μm)
Control (41)
SLD(26)
FMN2-/-(23)
h
Rab11a
MyosinVb tail
Merge
Rab11a
MyosinVb tail
i
Rab11a
MyosinVb tail-ActA
Merge
Rab11a
MyosinVb tail-ActA
j
p = 0.4213(ns)
p = 0.1711(ns)
Endosome speed (μm/s)
Control (10)
MyosinVb tail (9)
MyosinVb tail-ActA(10)
k
Mitochondria
MyosinVb tail-ActA
Merge

Supplementary Figure 2. Quantification of mitochondrial distribution during and after GVBD and the mitochondria distribution in FMN2 ^{IRK}-expressed Fmn2$^{-/-}$ oocytes and LatA-treated WT oocytes.

a, Schematics of the method for quantifying mitochondria at spindle periphery and mitochondria outside spindle periphery. **b**, Quantification of mitochondria distribution during and after GVBD. Green curve: quantification of mitochondria intensity at spindle periphery. Red curve: quantification of mitochondria intensity outside spindle periphery. Time 0 shows a GV oocyte, and GVBD starts around 30 min. **c,** Representative images showing that expression of FMN2^{IRK} did not rescue mitochondria distribution in Fmn2$^{-/-}$ oocytes. *n* = 28. Scale bar, 20 µm. **d,** Representative images showing that LatA treatment causes mitochondria to be dispersed from the spindle periphery. *n* = 39 (Control); *n* = 40 (LatA). Scale bar, 20 µm. **e,** Quantification of mitochondria accumulation around the spindle (mitochondria at spindle periphery/mitochondria outside spindle periphery) in LatA-treated oocytes. Data are represented as mean ± SD, from 3 independent experiments. Two-tailed unpaired t-test. Oocyte numbers indicated in brackets. **f,** Two representative images of mitochondria distribution in SLD-expressing oocytes. *n* = 41. Scale bar, 20 µm. **g,** Quantification of mitochondrial cluster size in SLD-expressing and Fmn2$^{-/-}$ oocytes. One side ANOVA, Tukey's multiple comparisons test, Data are represented as mean ± SD, from 3 independent experiments. Oocyte numbers indicated in brackets. **h,** Representative images showing that Rab11a-labeled endosomes (white arrows) co-localized with Myosin Vb tail in MI oocytes. *n* = 9. Scale bar, 20 µm. Cropped images to the right showing endosomal structures at a higher magnification; scale bar, 5 µm. **i,** Representative images showing that Myosin Vb tail-ActA did not co-localize with Rab11-labeled endosomes (white arrows). *n* = 10. Scale bar, 20 µm. Cropped images to the right showing endosomal structures at a higher magnification; scale bar, 5

µm. **j,** Quantification of movement speed of Rab11-labeled endosomes in the control, Myosin Vb tail-expressing, or Myosin Vb tail-Acta-expressing MI oocytes. One side ANOVA, Tukey's multiple comparisons test. Data are represented as mean ± SD, from at least two experiments. Oocyte numbers indicated in brackets. **k,** A representative image showed that Myosin Vb tail-Acta was co-localized with mito-AcGFP. n = 10. Scale bar, 20 µm. Source data are provided as a Source Data file.

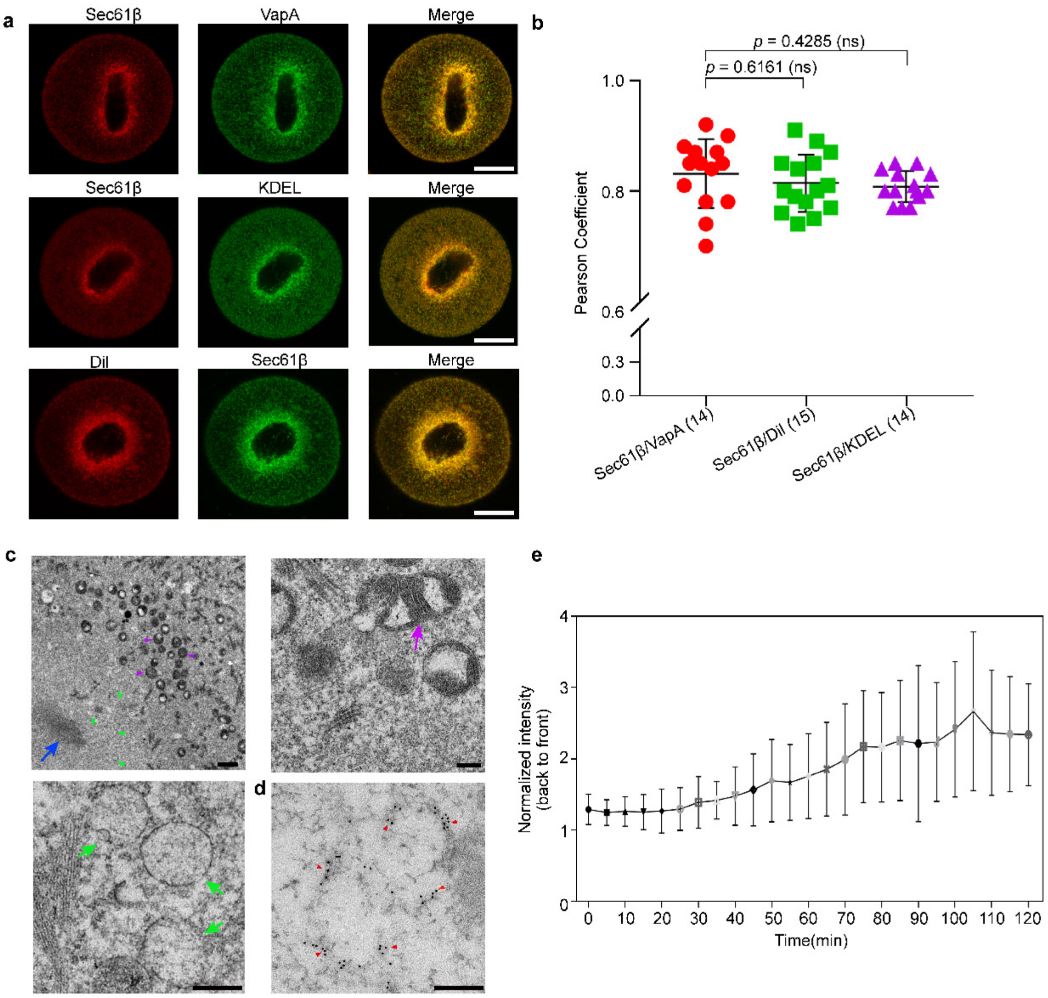

Supplementary Figure 3. Validation of Sec61β-labeled ER and mitochondria structures.

a, Representative images showing that Sec61β co-localizes with VapA, BFP-KDEL and DiI-labeled ER. Scale bar, 20 μm. **b,** Quantification of colocalization of Sec61β with VapA, BFP-KDEL and DiI. One side ANOVA, Tukey's multiple comparisons test. Data are represented as mean ± SD, from at least 2 independent experiments. Oocyte numbers indicated in brackets**. c,** Thin-sectioning EM of an oocyte in the metaphase of MI. magenta arrows point to mitochondria. Scale bar: 1 μm. Green arrows point to ER-like vesicles. Blue arrow point to chromosomes. n = 3.

The magnified images show the typical mitochondrial structure (right panel, n = 3) and ER-like vesicular structure (low panel, n = 3); scale bar: 0.2 μm. **d,** Immunogold labeling of Sec61β in mouse oocyte at the spindle migration stage. Sec61β localizes to the ER membrane, as detected by the immunogold particles (Red arrows). n = 5. Scale bar: 0.2 μm. **e**, Quantification of ER accumulation at the back edge of spindle during spindle migration (back/front). While there may be a trend for ER accumulation at the back edge of spindle during the spindle migration**,** the changes are not statistically significant. n = 5. One side ANOVA, Tukey's multiple comparisons test, p = 0.5385 (time point 0 vs time point 120). Source data are provided as a Source Data file.

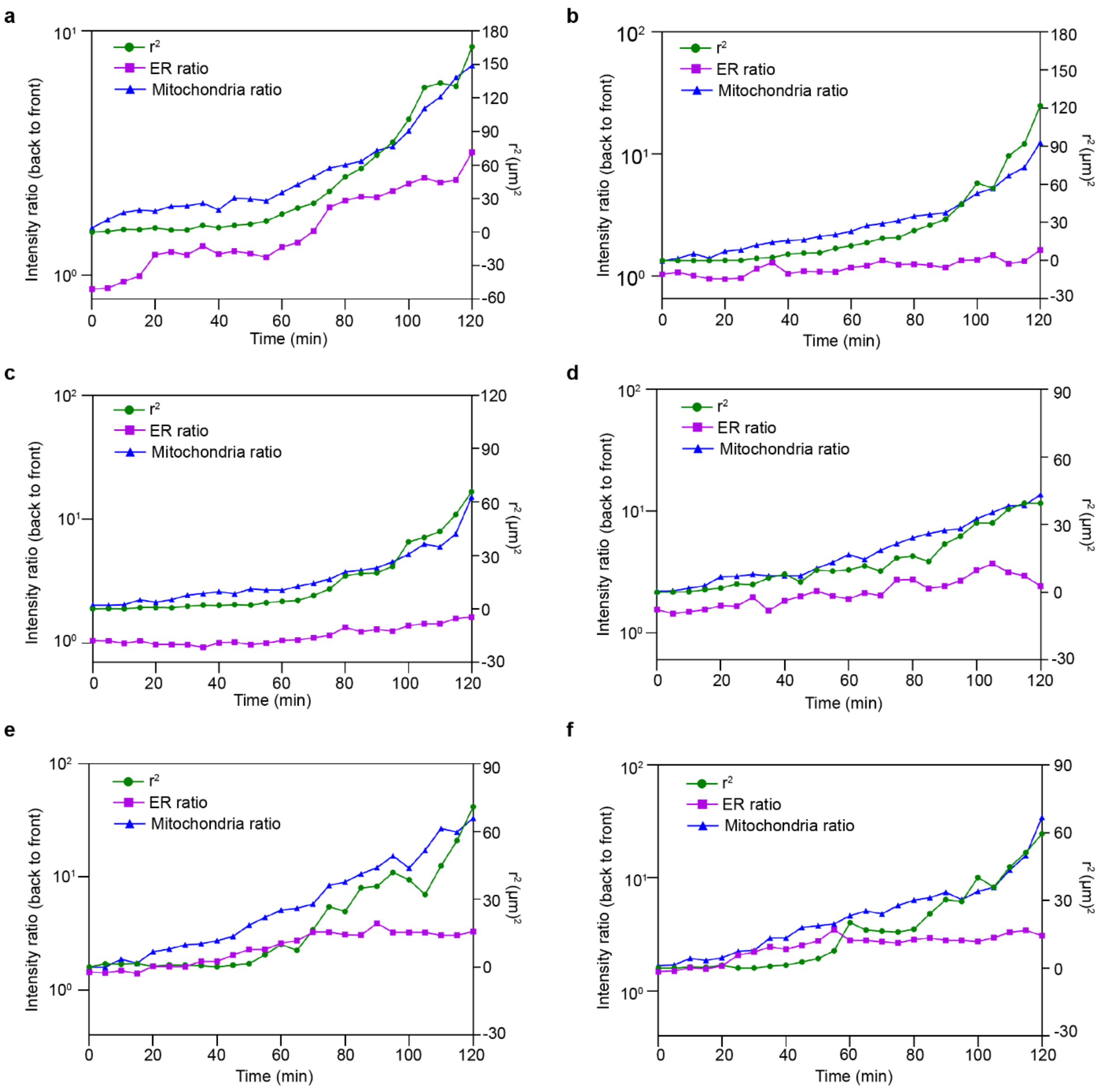

Supplementary Figure 4. Quantification of mitochondria and ER distribution as the back-to-front ratio along a 10 μm line crossing the long axis of spindle during the spindle migration in 6 individual oocytes (**a-f**). r^2 represented the squared displacement of the spindle (Green line); Blue line showed the mitochondria distribution as the back-to-front ratio during spindle migration; Magenta line showed the ER distribution as the back-to-front ratio during spindle migration. Source data are provided as a Source Data file.

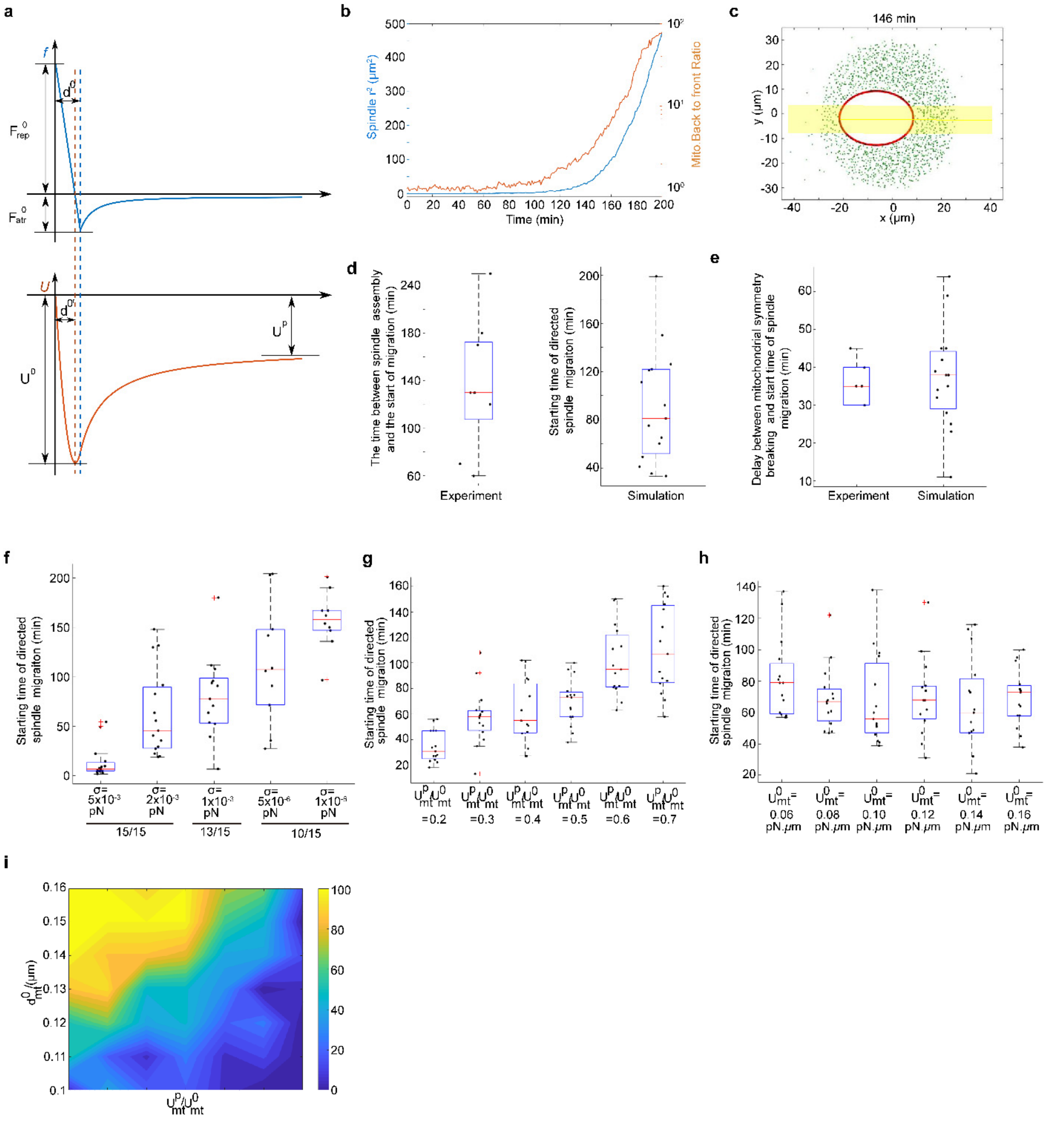

Supplementary Figure 5. The effect of different potential energy profiles on spindle migration.

a, Diagram of the attraction and repulsion forces and the potential energy. **b, c,** Polarized mitochondria distribution and the squared displacement of the spindle in the *x*-direction, $|x_S(t)$

$-x_s(0)|^2$ (**b**), quantified by taking the ratio of the number of Mitos at the back of the spindle to the number at the front within a 10 μm band along the long axis of the spindle, as shown in (**c**), during spindle migration. **d,** Box plots showing the time span between spindle assembly and the start of migration from experiments (n = 9) and simulations (n = 15) for control cases. **e**, Box plot showing the delay between mitochondrial symmetry breaking and the start time of spindle migration from experiments (n = 6) and simulations (n = 20). **f,** Box plots showing the effects of thermal fluctuation (σ) on the starting time for spindle migration. Directed spindle migration did not occur within the simulation time when σ = 0. When $\sigma = 5\times10^{-3}\text{pN}$ or $2\times10^{-3}\text{pN}$, 15/15 cells broke symmetry and initiated spindle migration within the simulation time; when $\sigma = 1\times10^{-3}\text{pN}$, 13/15 cells break symmetry; when $\sigma = 5\times10^{-4}\text{pN}$ or $1\times10^{-4}\text{pN}$, 10/15 cells break symmetry. n = 15 for all the conditions. $d_{\text{mt}}^{0\,\prime} = 0.10\,\mu\text{m}$, $U_{\text{mt}}^{0} = 0.12\,\text{pN}\cdot\mu\text{m}$, and $U_{\text{mt}}^{p} = 0.5U_{\text{mt}}^{0}$ were fixed. **g,** Effects of the ratio, $U_{\text{mt}}^{p}/U_{\text{mt}}^{0}$, on spindle migration while keeping the other parameters fixed. $d_{\text{mt}}^{0\,\prime} = 0.14\,\mu\text{m}$ and $U_{\text{mt}}^{0} = 0.16\,\text{pN}\cdot\mu\text{m}$. n = 15. All cells break symmetry within the simulation time. **h,** Effect of the depth of the potential well, $U^0{}_{\text{mt}}$, on spindle migration while keeping the other parameters fixed. $d_{\text{mt}}^{0\,\prime} = 0.14\,\mu\text{m}$ and $U_{\text{mt}}^{p}/U_{\text{mt}}^{0} = 0.5$. n = 15. All cells break symmetry within the simulation time. **d-h**, On each box, the central mark indicates the median, and the bottom and top edges of the box indicate the 25th and 75th percentiles, respectively. The whiskers extend to the most extreme data points not considered outliers, and the outliers are plotted individually using the '+' symbol. **i,** Contour of the percentage of cells/simulations that break symmetry by 90 min. Fixed $U_{\text{mt}}^{0} = 0.16\,\text{pN}\cdot\mu\text{m}$. Percentage is calculated from n = 15 simulations in each parameter set. Source data are provided as a Source Data file.

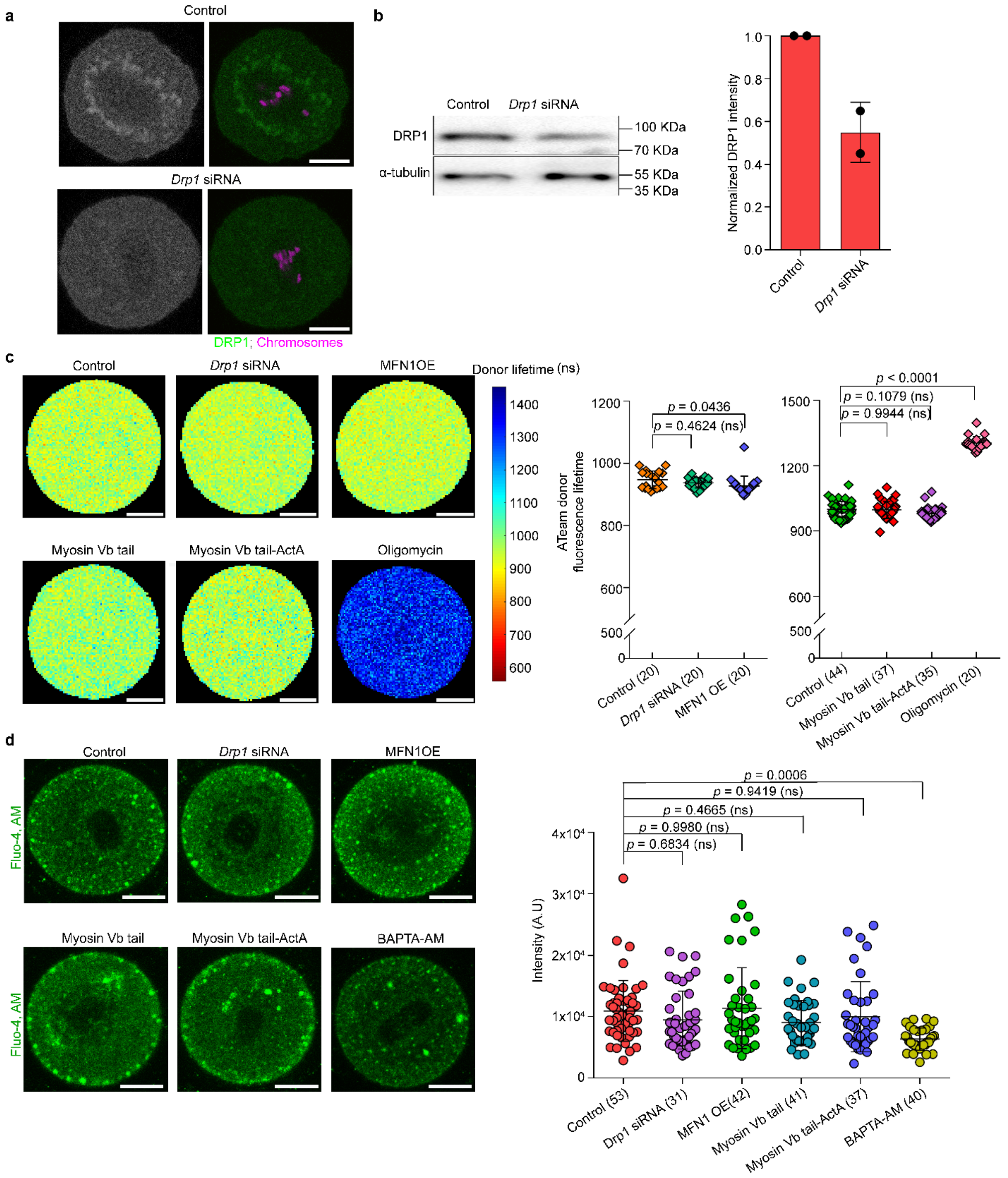

Supplementary Figure 6. Validation of *Drp1* knockdown with siRNA and the quantification of ATP content and Ca^{2+} concentration.

Immunofluorescence staining (**a**) and immunoblotting (*n* = 2) (**b**) using anti-DRP1 antibody showing that DRP1 level decreased after *Drp1* siRNA microinjection compared with that in the control siRNA-injected oocytes. Green, DRP1. Magenta, chromosomes. *n* = 8 (Control); *n* = 9 (*Drp1* siRNA). Scale bar, 20 µm. **c**, Representative FLIM images and quantification of ATeam donor fluorescence lifetime in un-treated control, oligomycin-treated, *Drp1* siRNA injected-oocytes, MFN1-expressing, Myosin Vb tail or Myosin Vb tail-ActA-expressing oocytes, from at least two independent experiments. One side ANOVA, Tukey's multiple comparisons test. Data are represented as mean ± SD. Oocyte numbers indicated in brackets. Scale bar, 20 µm. **d**, Representative images of intracellular free Ca^{2+} and quantification of Ca^{2+} concentration by using the calcium indicator Fluo-4 in BAPTA-AM-treated, *Drp1* siRNA-injected oocytes, MFN1-expressing, Myosin Vb tail or Myosin Vb tail-ActA-expressing oocytes, One side ANOVA, Tukey's multiple comparisons test. Data are represented as mean ± SD, from three independent experiments. Oocyte numbers indicated in brackets. Scale bar, 20 µm. Source data are provided as a Source Data file.

Uncropped immunoblots

a

Supplementary Figure 6b

b

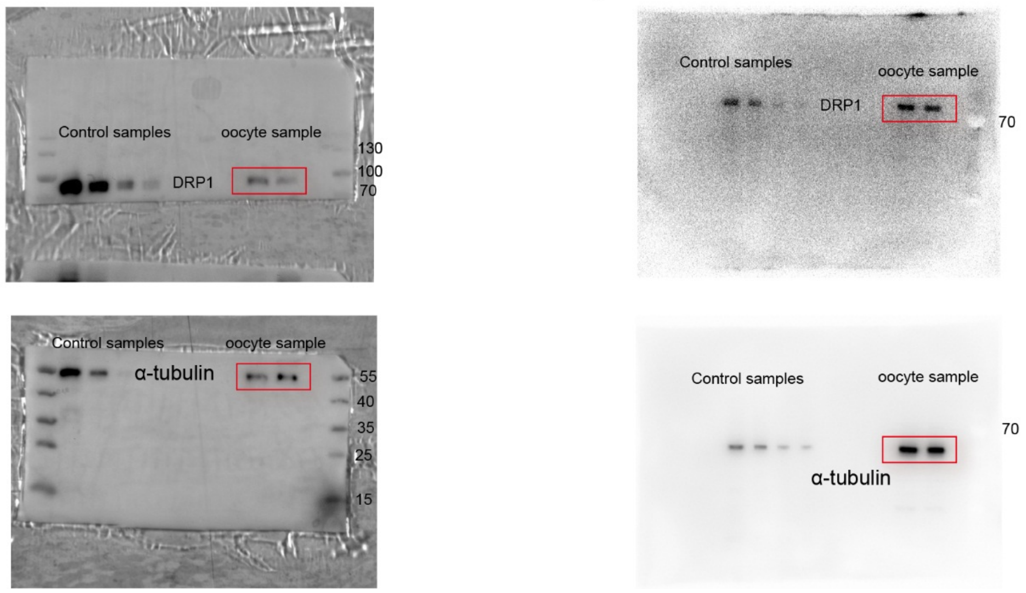

Supplementary Figure 7. Uncropped immunoblots in two individual experiments (a, b).

Supplementary Tables

Supplementary Table 1. A list of parameters used for the control case of the model.

Parameters	Description	Values	Sources
Δt	Time step in simulation	1 s	N/A
a	Long semi-axis of the spindle	15 μm	Observed
b	Short semi-axis of the spindle	11 μm	Observed
R_{mt}	Effective radius of a Mito	0.15 μm	Observed
N_{fa}	Number of actin filament bundles around the spindle	160	Estimated
N_{mt}	Total number of Mitos around the spindle	1440	Observed
η_{sa}	Drag coefficient along the long axis of the spindle	4×10^{-4} N·s·m^{-1}	Calculated (see text)
η_{sb}	Drag coefficient along the short axis of the spindle	8×10^{-4} N·s·m^{-1}	Calculated (see text)
η_{sr}	Drag coefficient of the rotation of the spindle	1×10^{-5}N·m·s	Estimated
η_{mt}	Drag coefficient of the Mitos	3×10^{-6} N·s·m^{-1}	Calculated (see text)
$l_{fa,max}$	Maximum length of an actin filament	0.35μm	Observed
δ	Effective radius of a G-actin	5 nm	Ref. 1
K_{on}	Maximum rate of actin polymerization	10 s^{-1}	Estimated
K_{off}	Maximum rate of actin depolymerization	3 s^{-1}	Estimated
F_{on}	Cut-off force for actin polymerization	50 pN	Ref. 2
F_{off}	Cut-off force for actin depolymerization	50 pN	Ref. 2
k_{am}	Coefficient of potential force between actin filament and Mitos	50pN·μm^{-1}	Estimated
d_{am}^{c}	critical distance of potential forces between Mitos and actin filaments	1 μm	Estimated
U^{0}_{mt}	Depth of the potential well	0.16 pN·μm	Estimated
U^{p}_{mt}	Plateau value of the potential profile	0.08 pN·μm	Estimated
$d^{0}_{mt}{}'$	Distance of the potential well to the origin	0.13μm	Estimated
$F^{0}_{rep,ms}$	Maximum repulsive force between Mitos and the spindle	25 pN	Estimated
$F^{0}_{atr,ms}$	Maximum attractive force between Mitos and the spindle	0.7pN	Estimated
d^{0}_{ms}	Critical distance for repulsion and attraction transition between Mitos and the spindle	0.1μm	Observed

Supplementary Table 2. List of primers used in this study.

Name	Sequence
POR1-mCherry FW	CTTCCGGATCCGGTTCAGGTATGGTGAGCAAGGGCGAGGAGGATAACATGGCC(OK805)
POR1-mCherry REW	CCGCGGCCGCTTACTTGTACAGCTCGTCCATGCCGCC (OK810)
pGEMHE-mCherry-Myosin Vb tail-Acta FW (for myosin Vb ail)	TAGCACAGGGGGCATGGATGAATTGTACAAGTCCACTAGTAGCTCCCCCGACAGCTACAG (OK23)
pGEMHE-mCherry-Myosin Vb tail-Acta REW (for myosin Vb ail)	CTGCACTTTTAGCAATTAGTTTGCTCCCACTCCCGCTTCCGACTTCATTGAGGAACTCCA (OK20)
pGEMHE-mCherry-Myosin Vb tail-Acta FW (for ActA)	GGAAGCGGGAGTGGGAGCAA (OK21)
pGEMHE-mCherry-Myosin Vb tail-Acta REW (for ActA)	GTACAGGATCCCGGGCCGTCGACTGCAGAATTCGAAGCTTTTAATTATTTTTTCTTAATT (OK17)
pGEMHE-mCherry-Mitofusin1 FW	CTAGCTCGAGATGGCAGAAACGGTATCTCCA (OK08)
pGEMHE-mCherry-Mitofusin1 REW	CAGAGGGCCCGCTGGGATTCTCCACTGCTCGGGTGTAGAA (OK09)
pGEMHE FMN2-SLD-EGFP FW	CCGCTCGAGCGGCACCATGGGAAGCCACTTGACCTCCGAGACACC (OK801)
pGEMHE FMN2-SLD-EGFP REW	CCGGAATTCCCAATGTCAGGATTCTACCTTCTTCTGTTGGG(OK802)
pGEMHE FMN2-CLD-EGFP FW	GATCTCGAGCACCATGGGGAACCAGGATGGGAAGCTGAAGAGAAGCGCAGGTGATGCCTCCCACG(OK799)
pGEMHE FMN2-CLD-EGFP REW	CCGGAATTCCAGGGTCAGCACACTCGGTATCCG(OK800)

References:

1. Cooper, Geoffrey M., Robert E. Hausman, and Robert E. Hausman. *The cell: a molecular approach*. Vol. 10. Washington, DC: ASM press, 2000.

2. Footer, M.J., Kerssemakers, J.W., Theriot, J.A. & Dogterom, M. Direct measurement of force generation by actin filament polymerization using an optical trap. Proc Natl Acad Sci U S A104, 2181-2186 (2007).
