# Supplementary material for: Dynamic organelle distribution initiates actin-based spindle migration in mouse oocytes
Source: Nat Commun. 2020 Jan 14;11:277. doi: 10.1038/s41467-019-14068-3 (PMC6959240; doi:10.1038/s41467-019-14068-3)
Supplement: Supplementary file 25 — Reporting Summary [file 41467_2019_14068_MOESM25_ESM.pdf]

nature research

Corresponding author(s): Rong Li

Last updated by author(s): Nov 3, 2019

Reporting Summary

Nature Research wishes to improve the reproducibility of the work that we publish. This form provides structure for consistency and transparency in reporting. For further information on Nature Research policies, seeAuthors & Referees and theEditorial Policy Checklist .

Statistics

For all statistical analyses, confirm that the following items are present in the figure legend, table legend, main text, or Methods section.

n/a	Confirmed	
☐	☒	The exact sample size (*n*) for each experimental group/condition, given as a discrete number and unit of measurement
☐	☒	A statement on whether measurements were taken from distinct samples or whether the same sample was measured repeatedly
☐	☒	The statistical test(s) used AND whether they are one- or two-sided<br>*Only common tests should be described solely by name; describe more complex techniques in the Methods section.*
☒	☐	A description of all covariates tested
☐	☒	A description of any assumptions or corrections, such as tests of normality and adjustment for multiple comparisons
☐	☒	A full description of the statistical parameters including central tendency (e.g. means) or other basic estimates (e.g. regression coefficient) AND variation (e.g. standard deviation) or associated estimates of uncertainty (e.g. confidence intervals)
☐	☒	For null hypothesis testing, the test statistic (e.g. *F*, *t*, *r*) with confidence intervals, effect sizes, degrees of freedom and *P* value noted<br>*Give P values as exact values whenever suitable.*
☒	☐	For Bayesian analysis, information on the choice of priors and Markov chain Monte Carlo settings
☒	☐	For hierarchical and complex designs, identification of the appropriate level for tests and full reporting of outcomes
☒	☐	Estimates of effect sizes (e.g. Cohen's *d*, Pearson's *r*), indicating how they were calculated

Our web collection on statistics for biologists contains articles on many of the points above.

Software and code

Policy information about availability of computer code

Data collection	Confocal images were acquired with Zeiss Zen for LSM780 and LSM800; western blot signals were detected by Bio-Rad Imager (Bio-Rad).The FLIM images were acquired with the use of Zeiss LSM 780 microscope and a PicoQuant system consisting of the PicoHarp 300 time-correlated single photon counting (TCSPC) module, two hybrid PMA-04 detectors, and Sepia II laser control module.
Data analysis	Statistical analysis was performed with Prism 8.0.1 (GraphPad Software, La Jolla, USA) and Microsoft office Excel (office 2016). Image J was used to handle the images. FLIM data were processed with SymPhoTime 64 v2.4 (PicoQuant) software. The computer codes were written by Wolfram Mathematica 11.3 and MATLAB R2018a and used for spindle migration, mitochondrial distribution analysis and the modeling of mitochondria vs spindle migration.

For manuscripts utilizing custom algorithms or software that are central to the research but not yet described in published literature, software must be made available to editors/reviewers. We strongly encourage code deposition in a community repository (e.g. GitHub). See the Nature Research guidelines for submitting code & software for further information.

Data

Policy information about availability of data

All manuscripts must include a data availability statement. This statement should provide the following information, where applicable:

- Accession codes, unique identifiers, or web links for publicly available datasets
- A list of figures that have associated raw data
- A description of any restrictions on data availability

Source data underlying Figs 1f-h, j-l, 2c-d, 3f-h, 3m, 4d, k-m, 5e, h and Supplementary Figs 2b, e, g, j, 3b, e, 4, 5b, d-i, 6c, d are provided as a Source Data file with the paper. All the relevant data supporting the finding of this study are available from the corresponding author upon request.The computer codes used for image analysis and the modeling of mitochondria vs spindle migration are publicly available on Github at https://github.com/RongLiLab/Duan-et.al.-2019.

Field-specific reporting

Please select the one below that is the best fit for your research. If you are not sure, read the appropriate sections before making your selection.

- [x] Life sciences
- [ ] Behavioural & social sciences
- [ ] Ecological, evolutionary & environmental sciences

For a reference copy of the document with all sections, see nature.com/documents/nr-reporting-summary-flat.pdf

Life sciences study design

All studies must disclose on these points even when the disclosure is negative.

Sample size	No predetermination of sample size were performed. The sample sizes (oocytes number for each experiment) were chosen based on the number of mature follicles of mice.
Data exclusions	No data was excluded from the studies.
Replication	All experiments were repeated on different days and all replicates showed consistent results.
Randomization	No randomization was performed because some of the parameters of modeling spindle migration is based on EM data.
Blinding	Investigators were totally blind towards the data sampling, but only mouse oocytes with a center located GV (germinal vesicle) were collected for experiment.

Reporting for specific materials, systems and methods

We require information from authors about some types of materials, experimental systems and methods used in many studies. Here, indicate whether each material, system or method listed is relevant to your study. If you are not sure if a list item applies to your research, read the appropriate section before selecting a response.

Materials & experimental systems

n/a	Involved in the study	
[ ]	[x]	Antibodies
[x]	[ ]	Eukaryotic cell lines
[x]	[ ]	Palaeontology
[ ]	[x]	Animals and other organisms
[x]	[ ]	Human research participants
[x]	[ ]	Clinical data

Methods

n/a	Involved in the study	
[x]	[ ]	ChIP-seq
[x]	[ ]	Flow cytometry
[x]	[ ]	MRI-based neuroimaging

Antibodies

Antibodies used	Rabbit monoclonal anti-DRP1(D6C7); Clontech; Cat# 8570;AB_10950498. Cell Signalling Technology<br>Anti-Rabbit IgG, HRP-linked Antibody;CST; Cat# 7074; AB_2099233. Cell Signalling Technology<br>Donkey anti-Rabbit IgG (H+L) Cross-Adsorbed Secondary Antibody, DyLight 550, # SA5-10039. Lot: RG2234352.<br>Rabbit-anti-Sec61β antibody, Cat# 07-205, Millipore,<br>Gold-conjugated goat anti–rabbit, Cat#111-205-144, AB_2338016. Jackson laboratories.
Validation	Rabbit monoclonal anti-DRP1(D6C7) has been validated by the Cell Signalling Technology (https://www.cellsignal.com/products/primary-antibodies/drp1-d6c7-rabbit-mab/8570).<br>Anti-Rabbit IgG, HRP-linked Antibody, validation by the Cell Signalling Technology (https://www.cellsignal.com/products/secondary-antibodies/anti-rabbit-igg-hrp-linked-antibody/7074).<br>Donkey anti-Rabbit IgG (H+L) Cross-Adsorbed Secondary Antibody has been validated by the manufacturer (https://www.thermofisher.com/antibody/product/Donkey-anti-Rabbit-IgG-H-L-Cross-Adsorbed-Secondary-Antibody-Polyclonal/SA5-10039).<br>Rabbit-anti-Sec61β antibody have been validated in previous publication from our lab (http://jcb.rupress.org/content/jcb/200/5/567.full.pdf?with-ds=yes).<br>Gold-conjugated goat anti–rabbit has been validated by Jackson laboratories (https://www.jacksonimmuno.com/catalog/products/111-205-144).

Animals and other organisms

Policy information about studies involving animals; ARRIVE guidelines recommended for reporting animal research

Laboratory animals	CF1 females: age 8-10 weeks; 129/Sv FMN2-/- mice: age 12-15 weeks;
Wild animals	The study did not involve wild animals.
Field-collected samples	The study did not involve field-collected samples.
Ethics oversight	All mice care and use were approved by the Institutional Animal Care and Use Committee at the Johns Hopkins University.

Note that full information on the approval of the study protocol must also be provided in the manuscript.
